# Botanical Description of British Plants

**Published:** 1808-09

**Authors:** 


### *Botanical Description of British Plants.*

[ Continued from our last, pp. 157—163. ]

24. LICHEN. *L. vulpinus.*

*Ang.* Wolf Lichen.

*Gen. Desc.* *As above.*

*Spec. Desc.* *Somewhat crustaceous; thread-like.* Plant lemon colour, upright, much branched, branches nearly of a length, angular; angles unequal; at first smooth, cylind. almost orange; paler with age, pitted, compressed, at length rough with yellow farinaceous powder. Shrubby, branches divided and subdivided, variously matted, 1 or 1 ½ inch long, cylind. thin, tender, in winter changing to dull-olive.—*In clusters round branches of trees, chiefly on oak.* BL. Jan. Dec.

*Use.* In Norway this plant is used to poison wolves, mixed with powdered glass, and strewed upon dead carcases. It dyes woollen yellow.—*Withering.*

25. LICHEN. *L. plicatus.*

*Ang.* Tree moss. Officinal stringy Lichen.

*Gen. Desc.* *As above.*

*Spec. Desc.* *Somewhat crustaceous, thread-like. Saucers* grey green, radiated, lateral and terminating, nearly flat, thin, brownish underneath, without any proper border but the edge fringed with radiating hairs. *Branches* pendant, thread-like, waved, matted, unequally subdivided; the slender divisions fibrous, rather stiff, grey. The old plants are covered with a rough, whitish warty crust. —*Branches of trees in thick woods, rare.* BL. Jan. Dec.

*Use.* It was formerly used in the shops as an astringent to stop hæmorrhages, and cure ruptures; but it has been laid aside by modern practice. We learn from Linnæus that it is made use of by the Laplanders as an application to the feet, for the relief of excoriations occasioned by much walking.—*Lightfoot.*

26. Lichen. *L. prunastri.*

*Gen. Desc.* *As above.*

*Spec. Desc.* *Leafy, herbaceous.* *Saucers* brown, white on the outside, on pedicles, browner with age. *Foliage* soft, broad, flat, bluish white; quite white and cottony underneath; pitted, rather upright: *warts* mealy.—*On trees.* Bl. Jan. Dec.

*Use.* This species has the remarkable property of imbibing and retaining odours; from hence it is made use of, as the basis of many perfumed powders.—*Withering.*

27. Lichen. *L. Islandicus.* *L. terestris.* *Lichenoides rigidum.*

*Ang.* Eatable Iceland Lichen. Eryngo-leaved Lichen.

*Gen. Desc.* *As above.*

*Spec. Desc.* *Leafy, herbaceous. Saucers* purplish brown, v. large, circular, entire, placed on the leaf. *Leaves* brown green, ascending, edges raised and fringed, crowded, connected, tough, membranous, several inches high, divided into irregular blunt lobes: *surface* smooth, shining, channelled, wrinkled, brown or pale green. *Substance* soft; horny, stiff when dry.—*Mountains, on the ground.*

*Use.* This plant is extremely mucilaginous; to the taste bitter and somewhat astringent: its bitterness as well as the purgative quality, which it manifests in a recent state, may be so far dissipated by drying, or extracted by a slight infusion in water, as that it may be converted into a tolerably grateful and nutritive article of food. One oz. boiled for a quarter of an hour, in a pint of water, yielded 7 oz. of as thick mucilage as that procured by a solution of gum arabic, 1 part in 3 of water. The Icelanders employ it in its fresh state as a laxative; but when deprived of this quality, and properly prepared, it is said to be an efficacious remedy in consumptions, coughs, dysenteries, and diarrhœas. Instances of its great success in consumptive disorders are related by many foreign medical writers, as well as of its efficacy in most of the above complaints. Dr. Herz says, that he found this so successful a medicine in dysentery, that since he first used it in that way, he never had occasion to employ any other remedy: it must be observed, however, that cathartics and emetics were always repeatedly administered before he had recourse to the Lichen, to which he occasionally added also opium. Dr. Crichton observes, that there are only two species of the Phthisis pulmonalis, where this sort of L. promises a cure, viz. phthisis hæmoptoica, and p. pituitosa, or mucosa. *See Med. Journ. v.* 10, *p.* 233. Lichen, no doubt, strengthens ens the digestive powers, and proves extremely nutritious, but the great medicinal efficacy attributed to it at Vienna, will not readily be credited at London.—It is commonly given in the form of a decoction; 1½ oz. boiled in one quart of milk; of this a tea-cup full is directed to be drank frequently in the course of the day. If milk disagree with the stomach, a simple decoction in water may be used; care should be taken that it be boiled over a slow fire, not longer than a quarter of an hour.—*Woodville.* This plant is used both for food and physic. In spring, a decoction of the fresh leaves in water, is used by the inhabitants of Iceland to purge away noxious humours, and it is said to act powerfully: when dried, the plant acquires a very different quality. They then grind it to powder, and eat it as common food, boiling it either in milk or broth, or making bread of it. Made into broth or gruel, it is said to be very useful in coughs and consumptions, for which purpose it is now much used, according to Haller and Scopoli, at Vienna.—*Lightfoot.* When it is boiled in broth by the Icelanders, or made into gruel to mix with milk, the first decoction is always thrown away, because it possesses a purgative quality.—*Withering.* For accounts of its efficacy in pulmonary consumption, *see Obs. on Pulm. Consump. or Ess. on Lichen Islandicus,* by Dr. Regnault of Paris, pub. 1802.

28. LICHEN. *L. pulmonarius.*

*Ang.* Lungwort. Hazel Rag. Hazel crottles, *N. of Ireland.* Rags, Herefordshire.

*Gen. Desc. As above.*

*Spec. Desc. Leafy, herbaceous. Saucers* red brown, mostly on the edges of the foliage, or in the hollows of the leaves, facing horizontally, circular, often 2 or 3 together; plants with saucers, rare, chiefly on the higher branches of trees. *Leaves* green, jagged, blunt, smooth; pitted; downy underneath, flat broad, loose, irregularly lobed. *Subst.* flexible, white and woolly within; *surface* green, bluish when dried, brown with age, spread over with net work. *Warts* mealy, crowded, on the edges of leaves, or net work.—*Trunks of trees; moist heaps of stones.* Bl. Jan. Dec.

*Use.* It has been reckoned very efficacious in consumptive cases, and from thence derives its trivial name: this opinion merits a further investigation.—Woollen cloth boiled in it, is dyed of a durable orange.—*Rutty.* It is used in Herefordshire for dying the stockings of the country people, to which it gives a good durable brown colour. *Withering.*

29. LICHEN.

29. Lichen. *L. caperatus.*

*Gen. Desc.* *As above.*

*Spec. Desc.* *Leafy, herbaceous.* *Saucers* red brown, rarely found, on the larger plants, either pale flesh colour, or the same colour as the leaves. *Foliage* pale green, wrinkled, waved at the edge, creeping. Not very leafy. Circular in its growth, from 1 inch to 1 foot in diameter; the small ones like a rose, the larger, not so regular. *Leaves* oblong, cut, terminating segments broadest; not pitted, marked with oblique unequal wrinkles.—*Stones, trees, holes.* Bl. Jan. Dec.

*Use.* In the North of Ireland, and on the Isle of Man, this is used to dye wool of an orange colour. Serge dyed with it becomes of a lemon colour, but if previously infused and boiled in urine, of a russet brown.—This is probably what the people in the North of Ireland call Stone-crottles, used to dye wool of an orange colour: it is also called Arcell, from the resemblance that it has to the Orchal in its use in dying.—*Rutty.*

30. Lichen. *L. pustulatus.*

*Gen. Desc.* *As above.*

*Spec. Desc.* *Root single, central,* stony. *Saucers* black, flattish, rare; only on the largest plants. *Fol.* grey brown, of a single leaf, concave, circular, slightly lobed, sprinkled with black bran-like powder; pitted underneath, 2 to 5 inches over, thin, membranaceous; lobes broad, shallow, deeper in old plants, covered with pustules, hollow, opening under the leaf. Plant brittle when dry; when wet, flexible, brown green at the edge, leaden grey in the centre, dirty yellow underneath. *Substance* white.—*Rocks with a south exposure.* Bl. Jan. Dec.

*Use.* From this species of Lichen a beautiful red colour may be prepared.—*Linn.* And it may be converted into an exceedingly black paint.—*Withering.*

31. Lichen. *L. caninus.*—*L. cinereus terrestris.*

*Ang.* Ash-coloured ground Liverwort.

*Gen. Desc.* *As above.*

*Spec. Desc.* *Foliage, leather-like.* *Tubercles* reddish brown, oblong, terminating. *Fol.* ash colour, mealy, creeping, lobes blunt, woolly and veined underneath: *leaves* a span long, 1 or 2 inches broad, widening as they grow out, lobes short, blunt, single or in strata, membranaceous, grey dull dirty green. *Targets* brown; underneath smooth, flesh colour; terminating, hard, solid, oblong but roundish.—*Roots, white fibres; grows on the ground in woods, heaths, stony places.* Bl. Jan. Dec.

*Use.*

*Use.* It has a faint weak smell, and a mucid sharp taste. It was long extolled for its singular virtue in preventing and curing that dreadful disorder which is produced by the bite of rabid animals. The pulvis antilyssus, which was first recommended as a preservative against rabies canina by Mr. Dampier, and was, by the authority of Sir Hans Sloane, published in the Phil. Trans. *(v.* 20, *p.* 49.*)* was composed of equal parts of this L. dried and pulverized, and of black pepper; but this medicine being found too hot, the powder was afterwards prepared of two parts L. to one of pepper. Dr. Mead, by whose desire this powder was in 1721 adopted in the London Pharmacopœia, declares that, in his experience, he had never known it to fail where it had been used, with the assistance of cold bathing before the hydrophobia came on. He directs the patient to be blooded to the extent of nine or ten ounces; afterwards 1 ½ drachm of the powder is to be taken in the morning fasting in half a pint of cow's milk warm, for four mornings successively: after these four doses are taken, the patient is directed to go into a cold bath every morning for a month, and then three times a week for a fortnight longer. On the character of Dr. Mead, the pulvis antilyssus was long retained in the Lon. Pharm. but on the revision of that book in 1788, it was deservedly expunged.—*Woodville.* In the History of the Royal Society, it is said that a dog became rabid, and bit several others belonging to the Duke of York; but by the timely administration of this Lichen, they were all preserved from madness. *Vol.* 492 *and v.* 3, 19. It has a disagreable musty taste; 1 ½ oz. of the leaves dried and pulverized, mixed with 2 drs. of black pepper powdered, compose the once celebrated pulvis antilyssus: but instances are known, where it has not prevented the hydrophobia.—*Lightfoot.*

32. Lichen. *L. apthosus.*

*Ang.* Green ground Liverwort with black warts.

*Gen. Desc. As above.*

*Spec. Desc. Fol. leather-like:* green, changing to brown, sprinkled with warts, lobes blunt, not veined underneath. *Tubercles* purple, or red brown, terminating, egg-shaped, crooked, warty, on short pedicles. *Surface* smooth, fine green when young, grey brown when old. *Warts* numerous, scattered, blackish. *Roots* v. long.—*Shady, stony, mossy places, and on rocks.* Bl. Jan. Dec.

*Use.* It is used by the country people to cure the thrush in children: they make an infusion of it in milk and give it to the child. In large doses, it is purgative and emetic, and and destroys worms.—*Withering.* In Upland it is made use of by the country people for the purpose: hence, from its curing the thrush or apthæ, it is named Apthosus. *Lightfoot.*

33. TREMELLA. *T. nostoc.*

*Ang.* Star-slough. Jelly rain. Tremella.

*Gen. Desc.* Substance gelatinous, transparent, uniform, lobed. Seeds dispersed through the jelly-like substance.

Obs. *It differs from the Gelatinous Lichens in having neither tubercles nor saucers.*

*Spec. Desc.* Olive-coloured, greenish or yellowish; plaited, waved. Sub-gelatinous, consisting of several leaves variously and lobed, waved, slightly adhering to the ground by a central root; substance very thin. Dark brown and brittle when dry. The seeds lie, like little strings of beads coiled up, within the folds of the plant, discoverable only by a microscope.—*Meadows and pastures after rain, on gravel walks frequent.* BL. Jan. Dec.

*Use.* It is recommended by Geoffroy as anodyne and vulnerary. A distillation of it, after being macerated for some time in water, is reputed to be an useful fomentation for pains in the joints: and a few grains of it powdered, taken internally and applied externally have been extolled in ulcerous cases, in cancers, and in the fistula; but it is to be feared on no good foundation.—This was termed by the ancient alchemists the flowers of heaven; and they entertained hopes of its proving an universal menstruum; but all their researches only shewed, that its constituent parts were a portion of phlegm oil, a urinous volatile salt, and a little fixed salt.—*Lightfoot.* This plant is supposed by the country people to be the remains of a meteor, or falling star. It has lately been asserted that it is of animal origin, but without sufficient reason. After very severe frosts, a gelatinous substance is often to be found, which might pass, on a careless observation, for a Tremella, but it is the remains of frozen frogs: this substance does not shrivel up in dry weather, as the Tremella does; nor is it plaited nor waved; and generally some of the bones of the frogs may be found in it: after the severe winter of 1789, Dr. Withering reports, that he found great quantities of these on the edges, and in the water of ponds.

( To be concluded in onr next. )

